# Effectiveness of resveratrol in inducing adeno-associated virus as a potential definitive therapy for SCN5A mutation in Brugada syndrome: a narrative review

**DOI:** 10.1186/s43044-025-00640-4

**Published:** 2025-05-08

**Authors:** Andin Zahrani Pateda, Andi Alika Azzahra, Kuni Zakiyyah Sumargo, Muchtar Nora Ismail Siregar

**Affiliations:** 1 Medical Study Program, Faculty of Medicine, State University of Gorontalo, Gorontalo, Indonesia; 2 Department of Cardiology and Vascular Medicine, State University of Gorontalo, Gorontalo, Indonesia

**Keywords:** Adeno-associated virus, Brugada syndrome, Resveratrol, SCN5A

## Abstract

**Objectives:**

Brugada syndrome (BrS) is a hereditary channelopathy that affects cardiac electrical signal transmission, with SCN5A gene mutation being the most common cause. Current BrS therapy primarily relies on Implantable Cardioverter Defibrillators, which are limited to arrhythmia prevention. Recent research has explored gene therapy as an alternative approach for managing BrS. Resveratrol, a non-ketone polyphenol compound, exhibits cardioprotective effects due to its antioxidant properties, which can influence gene expression through cellular signaling pathways, thereby modulating adeno-associated virus (AAV). This study aims to evaluate the effectiveness of resveratrol in enhancing the induction of AAV-based viral vectors as a potential definitive therapy for SCN5A mutations in BrS patients.

**Methods:**

A comprehensive literature search was conducted across multiple databases, including PubMed, Google Scholar, ScienceDirect, and PLOS ONE. The final stage involved assessing the eligibility of 47 studies, followed by a full-text review, which included seven studies for further analysis.

**Results:**

The findings indicate that this therapeutic approach highlights resveratrol’s crucial role as an activator of deacetylase proteins, influencing DNA repair processes, cell cycle regulation, and energy metabolism. Resveratrol facilitates the modulation of Voltage-Gated Calcium Channels, enabling calcium ion (Ca^2^⁺) influx into cardiomyocytes, thereby maintaining normal cardiac rhythm. Resveratrol enhances AAV-mediated gene delivery and expression through p53 pathway activation.

**Conclusion:**

Experimental studies have demonstrated that AAV-MOG1 gene therapy can restore sodium channel function, improve cardiac electrophysiological abnormalities, and ameliorate the clinical manifestations of BrS. Thus, resveratrol is potentially an inducer of AAV-mediated gene therapy for BrS.

## Introduction

Brugada syndrome (BrS) is a hereditary channelopathy recognized as a significant cause of sudden cardiac death, with an incidence of 1–5 per 10,000 individuals, accounting for 4–12% of total cases [1]. Southeast Asia is an endemic region for BrS, with case numbers reaching up to 14 times higher than the global prevalence, making it the leading cause of natural death in men under the age of 50 [2]. The prevalence among the Asian population is also significantly high, at 18 per 10,000 individuals, with male cases occurring 8–10 times more frequently than female cases, typically manifesting in the third or fourth decade of life [3].

Brugada syndrome (BrS) is associated with an increased risk of sudden death due to ventricular arrhythmias. The characteristic electrocardiographic findings, including ST-segment elevation of more than 2 mm with a coved-type morphology in more than one right precordial lead, reflect a channelopathy caused by an autosomal dominant genetic mutation. Various conditions, such as fever, vagal stimulation, medications, and electrolyte imbalances, can unmask the Brugada type-1 electrocardiographic pattern. Additionally, several reports have indicated that hypokalemia may trigger the Brugada electrocardiographic pattern [4].

Brugada syndrome is primarily caused by genetic mutations that disrupt cardiac electrical signal transmission, with SCN5A being the most commonly implicated gene [5]. More than 300 distinct mutations across 19 specific genes have been identified as potential contributors to BrS pathogenesis [2]. Approximately 60% of BrS cases are sporadic, with pathogenic mutations first discovered in the SCN5A gene, which encodes the α-subunit of the cardiac sodium channel (NaV1.5). Additionally, MOG1 (Multicopy suppressor of ts Gsp1), a small chaperone protein, has been found to facilitate NaV1.5 transport to the plasma membrane without affecting single-channel kinetics or conductance [6].

The implantation of an Implantable Cardioverter Defibrillator (ICD) remains the primary approach for preventing sudden arrhythmic death in BrS patients [5]. However, this intervention is limited to responding to arrhythmic episodes rather than preventing them outright [7]. Furthermore, ICDs are associated with adverse effects, including allergic reactions, infections, and inappropriate shocks. Given these limitations, innovative therapeutic approaches are needed to provide a more effective and targeted alternative for BrS management. Recent advances in gene therapy have introduced promising alternatives, mainly using viral vectors for targeted gene delivery. Among these, adeno-associated virus (AAV-MOG1) has emerged as a potential vector for delivering therapeutic genes to BrS-affected cells, specifically addressing SCN5A mutations responsible for sodium channel dysfunction. Unlike conventional therapies, gene therapy offers a transformative approach by correcting the underlying genetic defect rather than merely managing symptoms. The efficiency of AAV-mediated gene delivery can be enhanced by polyphenolic compounds, which mitigate barriers such as therapeutic gene instability caused by free radicals and other intrinsic and extrinsic factors.

Resveratrol, a polyphenolic compound, is considered a promising adjuvant to improve the effectiveness of AAV-MOG1-based gene therapy in BrS patients with SCN5A mutations [8]. Its anti-inflammatory properties increase viral vector tolerability and therapeutic efficacy [8]. Additionally, resveratrol is crucial in optimizing the microenvironment necessary for successful gene therapy, improving cardiomyocyte function by reducing oxidative stress and inflammation while enhancing cellular stability. This literature review aims to evaluate the potential effectiveness of resveratrol as a gene therapy adjuvant through AAV-MOG1 viral vector induction. Although resveratrol is expected to enhance cardiovascular outcomes in BrS patients synergistically, further research is required to elucidate its specific mechanisms of action in gene therapy [6].

## Methods

This narrative review is structured based on the analysis and synthesis of various references to evaluate the effectiveness of resveratrol in inducing adeno-associated virus (AAV) as a potential definitive therapy for SCN5A mutations in Brugada syndrome (BrS). A comprehensive literature search was conducted across multiple databases, including PubMed, Google Scholar, ScienceDirect, and PLOS ONE, in September 2024. The search began with title and abstract screening of studies identified within these databases. The initial search using the keywords “adeno-associated virus (AAV),” “Brugada syndrome,” AND “SCN5A” yielded 352 studies. After 121 duplicate studies were removed, the remaining articles underwent further reviewer screening. The next stage involved assessing the eligibility of 47 studies, followed by a full-text review, which included seven studies for further analysis.

**Fig. 1:**
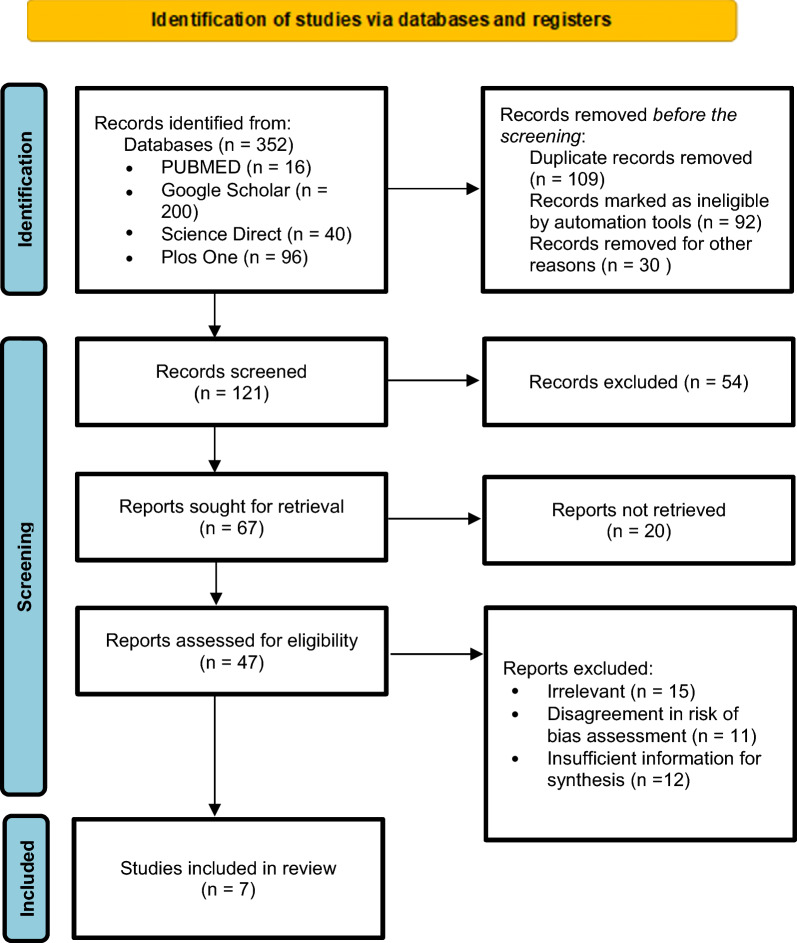
Review algorithm

This review includes only articles related to the application of gene-based therapy using the AAV model in Brugada syndrome. The inclusion criteria for this narrative review were as follows: The study population consisted of BrS patients, the study design was an original article, and the full-text was available. Only studies published within the past 10 years and written in Indonesian or English were considered. By analyzing these articles, this review aims to provide insights into the potential effectiveness of resveratrol as a compound capable of inducing AAV for SCN5A mutation therapy in BrS (Fig. 1).

## Results

### Pathogenesis of Brugada syndrome

In 1998, the first genetic mutation associated with Brugada syndrome (BrS) was identified as SCN5A, which encodes the alpha-subunit of the cardiac sodium channel Nav1.5 (α-subunit of the voltage-gated sodium channel) and is located on chromosome 3p21 [2]. Several studies have reported the association between mutations and BrS and modifier genes, most encoding sodium, potassium, calcium channels, or their associated proteins [9]. The primary pathophysiological mechanism underlying BrS is driven by SCN5A mutations, resulting in the loss of function (LoF) of the late cardiac sodium current (INa). The previous studies have also demonstrated that patients carrying SCN5A mutations experience more frequent episodes of syncope and more severe conduction abnormalities than those with other mutations [10]. Recent studies have identified three common genetic variants associated with Brugada syndrome (BrS) near the genes SCN5A, SCN10A, and HEY2. In total, over 20 genes have been linked to BrS. Thus far, there has not been a notable increase in rare genetic variants, except those related to SCN5A. The Clinical Genome Resource (ClinGen) has confirmed that SCN5A is the only gene with strong causal evidence for BrS. However, further investigation is necessary to understand how non-SCN5A genetic variants influence the long-term prognosis for individuals with BrS, particularly concerning conditions such as left atrial enlargement (LAE) and the risk of sudden cardiac death [10].

SCN5A is the most commonly associated gene with BrS, identified in approximately 20–25% of BrS patients. By 2010, nearly 300 cases of SCN5A mutations had been reported in BrS, including missense mutations, nonsense mutations, nucleotide insertions/deletions, and splice site mutations [2]. The number of SCN5A mutations continues to increase, and SCN5A remains the only undisputed genetic substrate for BrS [11]. The SCN5A gene plays a crucial role in the cardiac action potential’s rapid upstroke phase (phase 0). Pathogenic variations in this gene can cause sodium channel dysfunction, leading to slower conduction within the heart. Functional studies of these mutations have shown a loss of sodium channel function through various mechanisms. These include reduced expression of the sodium channel protein (Nav1.5) in the sarcolemma, the presence of non-functional channels, and changes in gating properties [9].

SCN5A encodes the Nav1.5 protein, a sodium channel (Na) family member, specifically subfamily 1, group 5. The lowercase “v” denotes voltage dependence, indicating that transmembrane voltage changes regulate channel activity [12]. SCN5A/Nav1.5 is highly expressed in the atrial and ventricular myocardium, His bundle, bundle branches, and Purkinje fibers, whereas its expression is low or undetectable in the sinoatrial and atrioventricular nodes [13]. Additionally, protein dysfunction contributes to channelopathies involving potassium, chloride, and calcium ion channels, which play a crucial role in cardiac depolarization and repolarization [14].

### Adeno-associated virus (AAV) in inducing SCN5A for Brugada syndrome

**Fig. 2:**
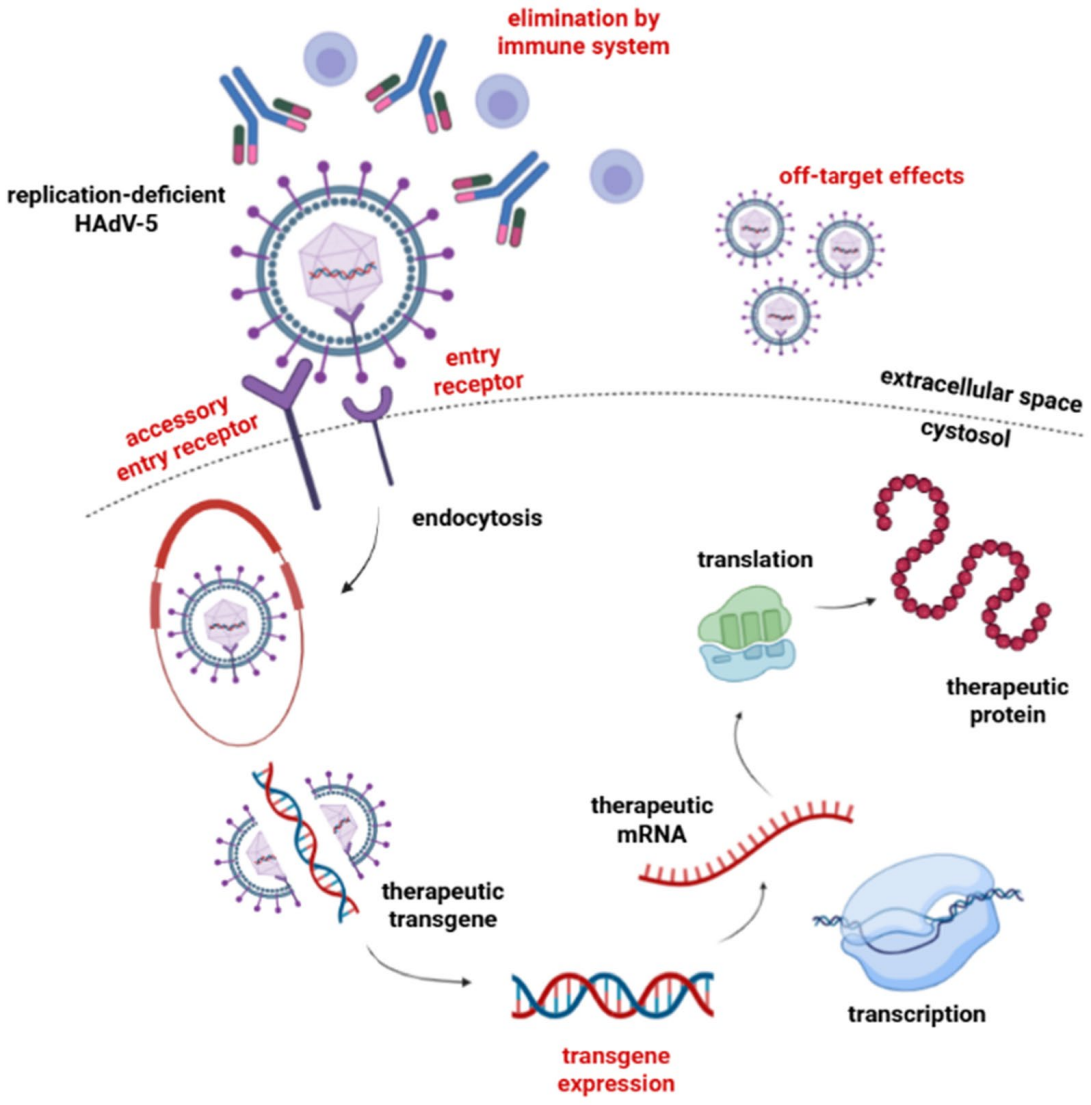
Mechanism of AAV gene expression as a viral vector and cardiovascular target cells (Created in https://BioRender.com) [16]

Various viral vectors have been demonstrated in vivo for gene therapy in the previous studies, with the most commonly used being adeno-associated viruses (AAV), adenoviruses (Ads), and lentiviruses (LV) [15]. Adeno-associated virus (AAV) is a non-pathogenic DNA virus utilized as a gene transfer vector. A study by Bongianino reported that AAV1, AAV6, and AAV9 could transduce the myocardium in mice, with AAV9 being the most cardiotropic agent. AAV has become the primary choice in clinical trials and applications approved by the US Food and Drug Administration (FDA). Long-term gene therapy requires a delivery vector to transport DNA or RNA into target cells, facilitating the introduction of foreign genetic material. These vectors are classified into viral and non-viral vectors. This review focuses on AAV9 as a viral vector due to its high efficiency in targeting cardiomyocytes. AAV9 is considered a promising vector for correcting genetic abnormalities affecting cardiac function, including Brugada syndrome (BrS), which is characterized by cardiac rhythm disturbances [15]. The selection of AAV as a viral vector for BrS gene therapy is based on several considerations, including its broad tissue tropism, relatively favorable safety profile, non-pathogenic nature, and minimal integration into the host cell genome. Additionally, AAV is known to maintain efficient transgene expression over the long term and enhance vector transduction efficacy. AAV has been widely used in gene therapy because it delivers genetic material into cells with minimal risk [16]. AAV-mediated gene induction is typically achieved through intravenous injection to facilitate the delivery of genetic material for therapeutic applications [17] (Fig. 2).

**Fig. 3:**
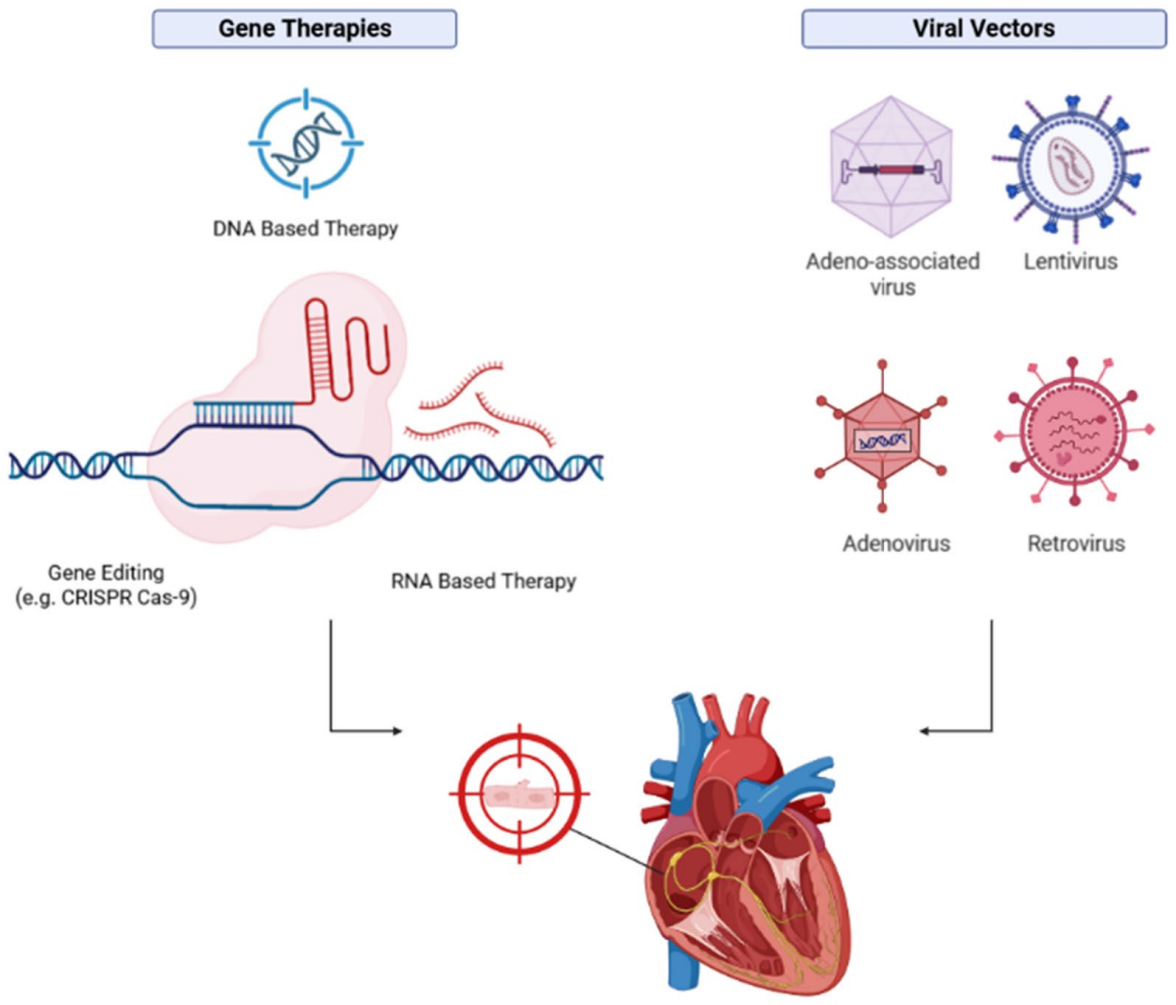
Combination of gene therapy and AAV delivery targeting cardiomyocytes (Created in https://BioRender.com) [20]

Adeno-associated virus (AAV) is considered a safe and efficient viral vector with long-term expression in target cells without causing significant damage to the host genome [18]. The elevation of troponin levels in Brugada syndrome (BrS) may indicate significant myocardial involvement, making troponin a valuable biomarker for monitoring cardiac conditions in BrS patients, particularly after treatments such as gene therapy using AAV. Measuring troponin levels can be an evaluation tool for AAV therapy by assessing its impact on myocardial viability and acting as a biomarker for treatment-induced damage [19]. Oxidative damage associated with AAV administration can be assessed through biomarkers such as malondialdehyde (MDA). In contrast, potential genetic damage due to AAV exposure can be evaluated by measuring reactive oxygen species (ROS) levels [20] (Fig. 3).

Using viral vectors in gene therapy presents a significant challenge in avoiding immune responses and the induction of pro-inflammatory cytokines and chemokines, which may lead to adverse effects. However, in studies involving AAV9 vector-based gene therapy in patients, there has been no clinical evidence of acute immune responses or massive inflammatory reactions following AAV administration, supporting the argument for its safety in gene therapy [21]. A study by Yu (2023) demonstrated that AAV9-MOG1 gene therapy in a BrS mouse model successfully reversed sodium channel defects, improved cardiac electrophysiological abnormalities, and ameliorated clinical manifestations of BrS. Nevertheless, comprehensive research is still required to thoroughly assess the therapy’s safety, as off-target effects remain a concern [22]. Cardiac action potentials are initiated when cardiomyocytes regulate voltage to facilitate sodium ion entry through sodium channels. These channels are crucial in myocardial cell excitability and optimal electrical conduction. The function of these sodium ion channels is also closely associated with the SCN5A mechanism in Brugada syndrome (BrS), mediated by Nav1.5, thereby playing a pivotal role in generating myocardial action potentials. Mutations in SCN5A lead to dysfunction in Nav1.5, resulting in either excessive or insufficient channel activity, which clinically manifests as cardiac arrhythmias [22].

A study by Yu (2023) found that MOG1 overexpression restored abnormal cardiac sodium current density in human-induced pluripotent stem cell-derived cardiomyocytes (hiCMs) transfected with the SCN5A-G1743R mutant plasmid. Similarly, MOG1 overexpression significantly increased INa density in hiCMs with the NaV1.5 p.D1275N mutant channel to levels comparable to those observed in hiCMs with wild-type (WT) NaV1.5 (*P* < 0.05) without affecting sodium channel activation or steady-state inactivation (*P* > 0.05). These findings suggest that MOG1 overexpression effectively reverses cellular abnormalities associated with the NaV1.5 p.D1275N mutation in human cardiomyocytes. Data from hiCM studies indicate that AAV9-MOG1 gene therapy may hold therapeutic potential for human patients [6].

### The anti-inflammatory potential of resveratrol in gene therapy for Brugada syndrome

Resveratrol is a non-ketone polyphenol compound with protective effects on the cardiovascular system. Various studies have demonstrated that resveratrol can prevent arrhythmias by regulating multiple ion channels, including Na⁺, K⁺, and Ca^2^⁺. Resveratrol has the potential to modulate calcium signaling pathways in excitable cells, particularly cardiomyocytes, highlighting its therapeutic role in managing abnormal cardiac rhythms associated with Brugada syndrome (BrS). Due to its antioxidant properties, resveratrol can influence gene expression through cellular signaling pathways that modulate the efficacy of adeno-associated virus (AAV) vectors. As an activator of Sirtuin (Sirt), a deacetylase protein involved in various cellular processes—including DNA repair, cell cycle regulation, and energy metabolism—resveratrol enhances the activity of the p53 pathway, which determines the effectiveness of AAV in delivering and inducing gene expression [23].

Resveratrol possesses anti-inflammatory properties that enhance the efficacy of gene therapy for BrS by inhibiting inflammatory mediators such as pro-inflammatory cytokines (IL-6, IL-8, and TNF-α) while promoting the release of anti-inflammatory cytokines [24]. A novel therapeutic strategy involving the combination of gene therapy and resveratrol aims to target oxidative stress and redox imbalance, offering a promising approach to improving treatment outcomes [25]. Therapeutic genes such as MOG1 function more effectively in a low-inflammation environment, facilitating NaV1.5 sodium ion channel function restoration. The anti-inflammatory effects of resveratrol may improve the success rate of gene therapy in BrS [26]. However, excessive antioxidant use can disrupt ROS-mediated cellular signaling, potentially leading to abnormal cell proliferation and increased oxidative stress by disturbing redox homeostasis, ultimately resulting in lipid, DNA, and protein damage [27].

The molecular mechanisms of resveratrol involve inhibiting pro-hypertrophic signaling, enhancing Ca^2^⁺ regulation in the myocardium, and activating pro-survival pathways (Akt-1 and GSK-3β) and stress signaling pathways (MKP-1). Additionally, resveratrol reduces oxidative stress and inflammation by suppressing iNOS activity, COX-2 expression, and ROS formation [28]. As an anti-inflammatory agent, resveratrol plays a crucial role in suppressing inflammatory responses through the mitogen-activated protein kinase (MAPK) pathway by inhibiting p65 phosphorylation and IκB proteins in the NF-κB signaling pathway, as well as p38 and ERK phosphorylation in the MAPK pathway, particularly in inflammatory conditions such as mastitis [29]. MAPK activation leads to signal translocation into the nucleus, phosphorylating various transcription factors, including Nrf2, NF-κB, and AP-1 [29]. The MAPK signaling pathway also plays a pivotal role in cell proliferation, differentiation, apoptosis, inflammation, and cellular responses to environmental stress, making it a potential target for various therapeutic strategies [30].

MAPK is a stress-induced kinase that includes c-Jun N-terminal kinase (JNK), extracellular signal-regulated kinase (ERK), Big MAP kinase (BMK), and p38 [31]. Among these, p38 MAPK is activated by pro-inflammatory stimuli, such as oxidative stress, ultraviolet (UVB) radiation, and inflammatory cytokines. Studies have shown that resveratrol can inhibit the activation of the ERK and p38 MAPK pathways induced by phorbol myristate acetate (PMA), suppressing COX-2 expression [29]. This inhibitory effect is likely mediated through the suppression of the p38 MAPK-cytosolic phospholipase A2-arachidonic acid-thromboxane A2-[Ca^2^⁺] cascade, as well as the activation of the nitric oxide (NO)/cyclic GMP pathway, which ultimately inhibits the activation of phospholipase C and/or protein kinase C (PKC) [31]. The inhibition of MAPK signaling pathways is thus considered a key mechanism contributing to the anti-inflammatory effects of resveratrol.

### The cardioprotective mechanism of resveratrol in Brugada syndrome

Resveratrol influences cellular signaling pathways, including activating Sirtuin 1 (Sirt-1) and AMP-activated protein kinase (AMPK), which regulate energy metabolism and cellular stress responses. Sirt-1 deacetylates multiple proteins, regulating genomic integrity, inflammatory responses, mitochondrial function, and resistance to oxidative stress reactions. Resveratrol therapy has been shown to reduce FOXO1 acetylation in myocardial tissue as a response to oxidative stress. The previous studies have indicated that oxidative stress induces the formation of aggressive free radicals, which disrupt normal cellular functions [32].

The cardioprotective effects of resveratrol stem from its ability to stimulate antioxidant enzyme production, such as catalase, superoxide dismutase, and glutathione peroxidase, ultimately reducing reactive oxygen species (ROS) levels [33]. Cardiovascular benefits of resveratrol have been observed in both in vitro and in vivo studies, demonstrating its ability to reduce ventricular arrhythmias and tachycardia while preventing cardiac remodeling [34]. Resveratrol exhibits antiarrhythmic properties by inhibiting L-type Ca^2^⁺ channels (L-type Cav) and activating slow K⁺ channels (IKs), as well as increasing K⁺ currents mediated by ATP-sensitive K⁺ channels (KATP). These effects contribute to stabilizing cardiac cell membranes and preventing arrhythmias. Resveratrol exhibits antiarrhythmic effects by prolonging the cardiac refractory period, achieved through inhibiting Na⁺ channels and transient and sustained K⁺ currents, thereby suppressing ventricular arrhythmias. Additionally, this effect is mediated by the modulation of late Na⁺ currents (INaL) via the upregulation of Na⁺/Ca^2^⁺ exchanger (NCX) activity, which plays a crucial role in regulating intracellular diastolic Ca^2^⁺ concentrations in ventricular myocytes. In vivo studies have demonstrated that resveratrol effectively reduces ventricular arrhythmias and tachycardia induced by coronary artery ligation, enhances survival rates, and inhibits cardiac remodeling in myocardial infarction models [35].

Beyond its cardiac benefits, resveratrol also contributes to vasodilation by enhancing nitric oxide (NO) synthesis, which is associated with Ca^2^⁺ concentration regulation in endothelial and vascular smooth muscle cells. In this process, resveratrol inhibits intracellular Ca^2^⁺ release from the sarcoplasmic reticulum through the ryanodine receptor (RyR) and inositol 1,4,5-trisphosphate receptor (IP3R) pathways, reduces troponin-C sensitivity to Ca^2^⁺, and enhances cardiomyocyte responsiveness. These effects position resveratrol as a promising therapeutic approach for managing Brugada syndrome (BrS) arrhythmias [36]. Additionally, resveratrol plays a crucial role in enhancing the efficacy of gene therapy in BrS by modulating Voltage-Gated Calcium Channels (VGCC). VGCCs enable the influx of Ca^2^⁺ into cardiomyocytes, which is crucial for initiating contraction and sustaining a normal cardiac rhythm. These channels are categorized into high-voltage-activated (HVA) channels, including L-type (CaV1) and P/Q-type (CaV2), as well as low-voltage-activated (LVA) channels, such as *T* type (CaV3). Dysregulation of these channels may lead to elevated cytosolic calcium levels, contributing to hyperexcitability and arrhythmias [37].

## Conclusion

Brugada syndrome (BrS) is an electrical transmission disorder of cardiac sodium ion channels, primarily caused by genetic mutations, with SCN5A being the most commonly affected gene. Gene therapy remains an innovative treatment strategy under development for this disease. The therapeutic approaches to address BrS require comprehensive research to target BrS-associated genetic mutations selectively. Resveratrol, with its antioxidant properties mediated through oxidative reactions and sodium channel modulation, has demonstrated potential as an adjuvant therapy to enhance the efficacy of gene therapy for BrS. The innovative application of resveratrol in adeno-associated virus (AAV) induction as a potential adjuvant holds promise as an alternative therapeutic strategy. However, further research is required to explore its potential, safety, bioavailability, and precise cellular targeting. A deeper understanding of these factors is essential to establish resveratrol-based biomolecular innovations as a definitive therapy aimed at reducing BrS-related morbidity and mortality rates.

## Data Availability

No datasets were generated or analyzed during the current study.

## Abbreviations

AAV: Adeno-associated virus
AAV-MOG1: Adeno-associated virus multicopy suppressor of Gsp1
Ads: Adenoviruses
AV: Atrioventricular
BMK: Big MAP kinase
BrS: Brugada syndrome
ERK: Extracellular signal-regulated kinase
FDA: Food and Drug Administration
GWAS: Genome-wide association studies
HiCMs: Human-induced pluripotent stem cell-derived cardiomyocytes
HVA: High-voltage-activated
ICDs: Implantable cardioverter defibrillators
IP3R: Inositol 1,4,5-trisphosphate receptor
JNK: C-Jun N-terminal kinase
LAE: Left atrial enlargement
LOF: Loss of function
LV: Lentiviruses
LVA: Low-voltage-activated
MAPK: Mitogen-activated protein kinase
MDA: Malondialdehyde
NCX: Na⁺/Ca^2^⁺ exchanger
NO: Nitric oxide
PKC: Phospholipase C and/or protein kinase C
PMA: Phorbol myristate acetate
ROS: Reactive oxygen species
RyR: Ryanodine receptor (Ryr)
SA: Sinoatrial
SCN5A: Sodium voltage-gated channel alpha-subunit 5 gene
Sirt: Sirtuin
UBV: Ultraviolet radiation
VGCC: Voltage-gated calcium channels
